# Image-Based CFD Analysis of Blood Flow in Non-Porous and Porous Prosthetic Vascular Grafts

**DOI:** 10.3390/bioengineering13080915

**Published:** 2026-08-13

**Authors:** Labin Kim, Suk-Hee Park, Seong Hoon Jeong, Yeong-Seo Kim, Ju Ran Kim, Kyung Eun Lee

**Affiliations:** 1 Department of Mechanical Engineering, Inha University, 100 Inha-ro, Michuholgu, Incheon 22212, Republic of Korea; 2 School of Mechanical Engineering, Pusan National University, Busan 46241, Republic of Korea; 3 Advanced Textile R&D Department, Korea Institute of Industrial Technology (KITECH), Ansan 15588, Republic of Korea; 4 Biohybrid Systems Research Center, Inha University, 100 Inha-ro, Michuhol-gu, Incheon 22212, Republic of Korea

**Keywords:** blood flows, porosity, prosthetic graft, hemodynamics, end-to-end anastomosis

## Abstract

A prosthetic vascular graft is an important vascular conduit used for end-to-end anastomosis; however, optimizing pore morphology to improve local hemodynamic performance remains a significant challenge. The purpose of this study was to investigate the influence of regular and reconstructed irregular pore morphologies on local blood flow characteristics in prosthetic vascular grafts. Three graft models, including a non-porous model, a regular porous model with regular square-prism pores, and a reconstructed irregular porous model reconstructed from segmented scanning electron microscopy (SEM) images, were employed to simulate blood flow within virtual end-to-end anastomoses. To enable a direct comparison of pore morphology, the regular and irregular porous models were designed with the same porosity. Steady laminar blood flow through virtual end-to-end anastomoses was simulated using computational fluid dynamics (CFD) assuming Newtonian blood, rigid vessel walls, and a no-slip boundary condition. The non-porous model exhibited a flow pattern representative of a conventional graft and served as the baseline for comparison. Compared with the regular porous model, the reconstructed irregular porous model generated more irregular recirculation patterns, a non-uniform pressure distribution, and significantly greater spatial heterogeneity in wall shear stress (WSS). Although the irregular porous model exhibited the lowest mean and median WSS, it produced the highest localized WSS at sharp pore edges and the largest surface area exposed to low WSS. Quantitative analyses demonstrated that the irregular pore morphology increased the spatial variability in WSS and altered the distribution of low-WSS regions and local recirculation compared with the regular porous model under the same porosity conditions. The results demonstrate that pore morphology, rather than porosity alone, significantly influences local hemodynamic characteristics under identical porosity conditions. Furthermore, the SEM-based reconstruction approach captured realistic spatial variations in flow and WSS that could not be fully reproduced by an idealized regular porous model. These findings provide insights into the hemodynamic effects of porous graft architectures and may contribute to the design and optimization of prosthetic vascular grafts.

## 1. Introduction

Prosthetic vascular grafts play a crucial role in vascular reconstruction and bypass procedures by providing an alternative conduit for blood flow when native vessels become diseased or unavailable. Synthetic grafts such as expanded polytetrafluoroethylene (ePTFE) and polyethylene terephthalate (Dacron) have been widely used in clinical practice for several decades. Despite continuous improvements in biomaterials, graft manufacturing technologies, and surgical techniques, prosthetic grafts remain susceptible to complications, including thrombosis, intimal hyperplasia, restenosis, and eventual graft failure [1,2,3,4,5]. These complications continue to limit long-term graft patency and represent a major challenge in vascular surgery.

A growing body of clinical and experimental evidence has demonstrated that local hemodynamic conditions play a critical role in the initiation and progression of graft-related complications. In particular, wall shear stress (WSS), flow separation, recirculation zones, and oscillatory flow patterns have been shown to influence endothelial cell function, platelet activation, thrombus formation, and vascular remodeling processes [6,7,8,9,10,11,12]. Regions exposed to low WSS and disturbed flow are frequently associated with neointimal hyperplasia and thrombotic events, ultimately leading to restenosis and graft occlusion. Consequently, understanding the interaction between prosthetic graft structures and blood flow has become essential for improving graft performance and long-term clinical outcomes.

Among the various structural characteristics of prosthetic vascular grafts, the porous nature of the graft wall has attracted considerable attention. In commercial grafts such as ePTFE and Dacron, porous microstructures are intentionally incorporated to provide flexibility and compliance. Since implanted grafts are continuously subjected to pulsatile pressure, bending, and deformation within the physiological environment, a certain degree of structural porosity is required to accommodate these mechanical demands. The interconnected pore network formed between fibers allows the graft wall to deform more readily, thereby improving its flexibility and mechanical compatibility with native vessels. Consequently, porosity is not merely a byproduct of the manufacturing process but an essential structural feature that contributes to the mechanical functionality of prosthetic grafts.

In addition to its mechanical role, the morphology of graft pores can significantly influence the local hemodynamic environment. Unlike non-porous grafts, porous grafts contain interconnected surface pores that modify the local blood flow interaction between the flowing blood and the graft wall. Such porous structures can alter near-wall flow behavior, modify wall shear stress distributions, and influence local flow structures adjacent to the graft wall. Furthermore, previous experimental studies have suggested that porous graft surfaces may promote tissue integration and endothelialization following implantation [13,14,15,16,17,18,19,20,21,22]. Therefore, the morphology of individual pores is expected to play an important role in determining the local hemodynamic characteristics of prosthetic vascular grafts.

Previous investigations have primarily focused on the global hemodynamic behavior of vascular grafts without explicitly considering the detailed morphology of graft pores [10,21,22,23]. Early studies established the importance of wall shear stress in the development of intimal hyperplasia and graft failure, providing a fundamental understanding of the relationship between altered flow dynamics and vascular pathology. More recently, several researchers have investigated the effects of graft porosity using experimental and computational approaches [22,23,24,25,26]. In many numerical studies, porous graft walls were represented using homogenized porous-medium models based on Darcy-type formulations, in which the detailed pore geometry was replaced by an equivalent permeability parameter [24]. Such approaches have provided valuable insights into the overall hemodynamic effects of graft porosity. However, these methods inherently neglect the realistic morphology of individual pores and the complex microscale blood–surface interactions occurring within the porous structure.

Although numerous studies have investigated porous vascular grafts, most computational models have represented graft porosity using idealized pore geometries or homogenized porous-medium formulations [10,21,22,23]. Consequently, the direct influence of realistic irregular pore morphology on blood flow dynamics remains poorly understood. To the authors’ knowledge, few studies have reconstructed actual graft microstructures from scanning electron microscopy (SEM) images and systematically compared the resulting hemodynamic characteristics with those of non-porous and regular porous graft models. This gap motivates the present study.

The limitations of existing studies become particularly significant when considering the actual microstructure of commercially available prosthetic grafts [22]. In reality, graft surfaces consist of highly irregular pore architectures characterized by substantial variations in pore size, shape, orientation, and spatial distribution. These morphological features arise from manufacturing processes and can significantly influence local flow structures, microscale recirculation, and wall shear stress distributions. Consequently, the hemodynamic characteristics of reconstructed irregular porous grafts may differ substantially from those predicted by idealized pore models or homogenized porous-medium approximations.

Recent advances in scanning electron microscopy and image-based reconstruction techniques have created new opportunities to overcome these limitations. High-resolution SEM images enable detailed characterization of graft microstructures, while image-based modeling methods allow realistic pore geometries to be directly incorporated into computational fluid dynamics (CFD) simulations. Such approaches make it possible to investigate blood flow behavior around realistic pore geometries and capture microscale hemodynamic phenomena that cannot be resolved using conventional porous-medium formulations.

Therefore, the primary objective of this study is to present an image-based methodology for reconstructing realistic porous graft geometries from SEM images of actual prosthetic vascular graft surfaces and to investigate their hemodynamic characteristics using CFD simulations. Specifically, blood flow behavior in a non-porous graft, a regular porous graft, and a reconstructed irregular porous graft with the same porosity is compared to evaluate the influence of pore morphology on velocity fields, near-wall flow structures, and wall shear stress distributions.

The novelty of the present work lies in the direct incorporation of SEM-derived reconstructed irregular pore morphologies into CFD simulations, enabling a realistic representation of graft microstructures and their effects on blood flow. Unlike previous studies that relied on homogenized porous-medium models or simplified pore geometries, the present study aims to resolve microscale flow structures around individual pores and evaluate their influence on the surrounding flow field.

We hypothesize that reconstructed irregular pore morphologies generate local hemodynamic characteristics that differ from those of regular pore geometries because of their complex pore architecture and the resulting microscale flow structures. By elucidating these mechanisms, the present study seeks to provide new insights into the design and optimization of prosthetic vascular grafts.

## 2. Methods

### 2.1. Geometry

This work focuses on the simulation of blood flow in prosthetic grafts with different pore morphologies. Three types of prosthetic grafts were modeled, including a non-porous rigid wall (Model I), a regular porous wall with square-prism pores (Model II), and a reconstructed irregular porous wall generated from segmented scanning electron microscopy (SEM) images (Model III). To enable a direct comparison of pore morphology, Models II and III were designed with the same porosity (59%). These numerical models were used to investigate the influence of pore morphology on local hemodynamic characteristics and blood flow within prosthetic grafts.

**Figure 1:**
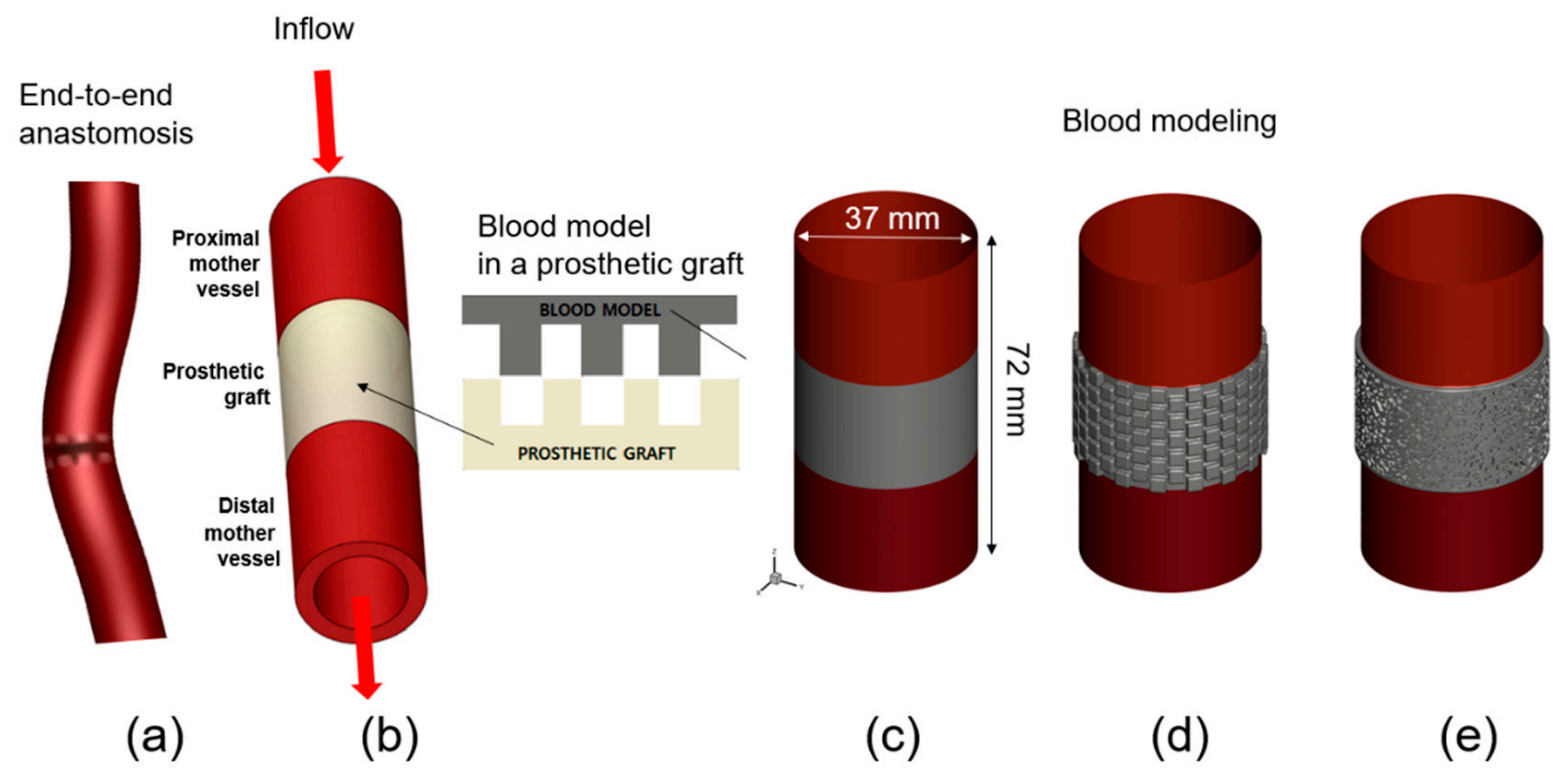
Schematic illustration of end-to-end anastomosis created by grafting a prosthesis. (**a**) End-to-end anastomosis. (**b**) The schematic diagram of a mother vessel with a prostheshetic vascular graft. (**c**) Model I: blood flow model of a non-porous graft. (**d**) Model II: blood flow model of a regular porous graft (59% porosity). (**e**) Model III: blood flow model of a reconstructed irregular porous graft (59% porosity).

A schematic drawing of the prosthetic grafts for end-to-end anastomosis is shown in Figure 1a,b. End-to-end anastomosis models were constructed using three types of prosthetic grafts positioned between the proximal and distal mother vessels, as shown in Figure 1c–e. All blood models in three grafts had a lumen diameter of 37 mm and a length of 72 mm (Figure 1c–e). The lengths of the proximal mother vessel, the prosthetic graft, and the distal mother vessel were identical at 24 mm (Figure 1c–e).

Model I represents blood flow in a straight tube-shaped graft with a non-porous rigid wall, as depicted in Figure 1c. As illustrated in Figure 1d, Model II represents blood flow through a graft with regular square-prism pores.

**Figure 2:**
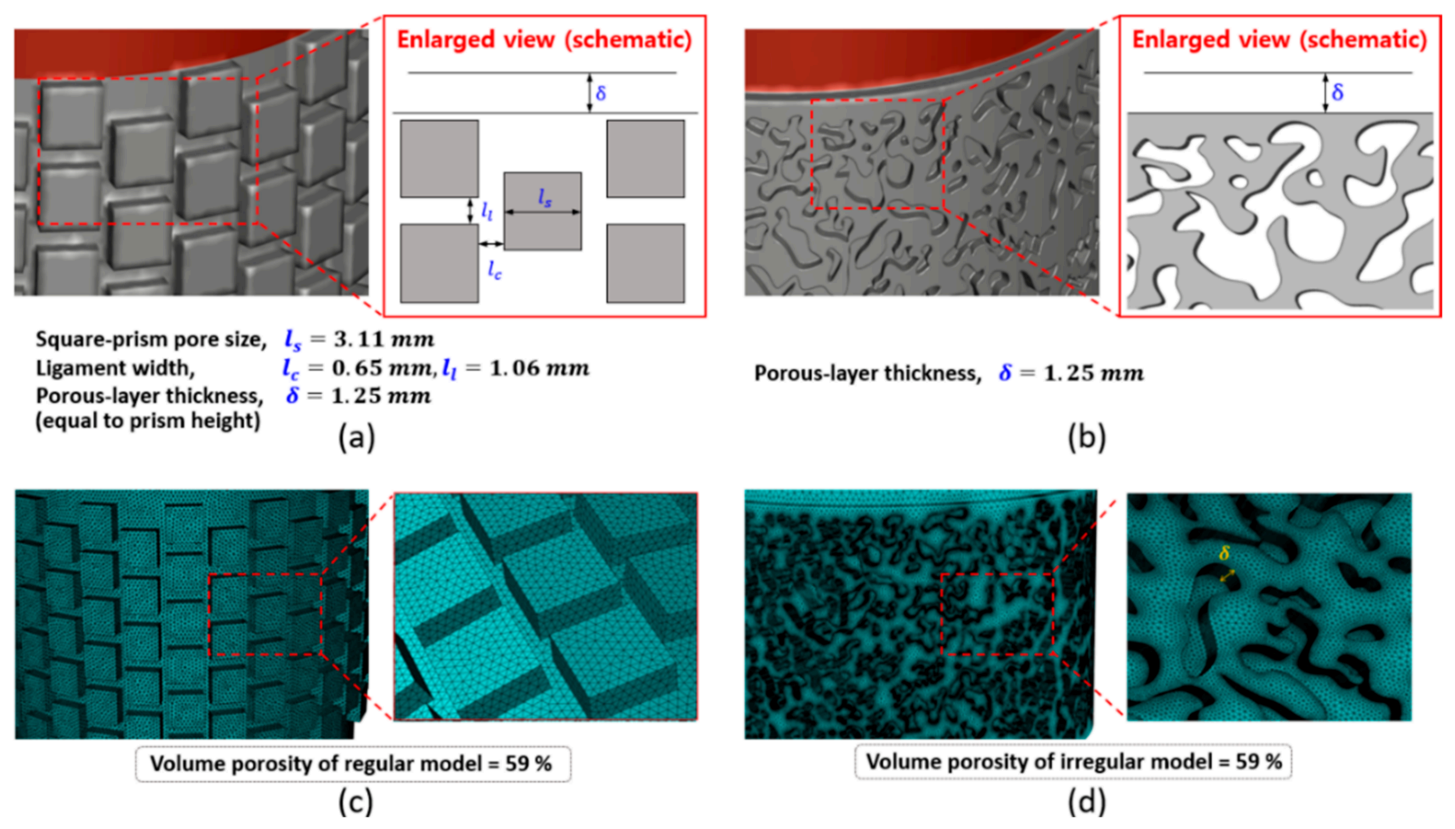
Pore morpologies of Models II and III. (**a**) Model II: regular squre-prism pores in the prosthetic graft (square-prism pore size = 3.11 mm × 3.11 mm; porous-layer thickness = 1.25 mm; porosity = 59%). (**b**) Model III: reconstructed irregular realistic pores in prosthetic graft (porous-layer thickness = 1.25 mm; porosity = 59%). (**c**) Computational meshes of Model II. (**d**) Computational meshes of Model III.

Model II consists of a straight tube-shaped graft incorporating a regular porous layer with square-prism pores over a 24 mm long central section. The regular porous layer consists of square-prism pores with a pore size of 3.11 mm ${\;(l}_{s})$ × 3.11 mm ($l_{s})$ and a porous-layer thickness (δ) of 1.25 mm, as shown in Figure 2a,c. The square-prism pores are regularly arranged in both the circumferential and longitudinal directions, with ligament widths ($l_{c})$ of 0.65 mm and ($l_{l})$ of 1.06 mm, respectively (Figure 2a,c). The pores are isolated from one another without interconnections. The volume porosity (void volume fraction) of the porous layer was set to 59%, identical to that of Model III, to enable a direct comparison of pore morphology.

Model III, shown in Figure 1e, represents blood flow through a graft with a reconstructed irregular porous structure generated from segmented scanning electron microscopy (SEM) images. And the porous-layer thickness (δ) was 1.25 mm (Figure 2b,d). The detailed reconstruction procedure is described in Section 2.2.

### 2.2. Reconstruction of the SEM-Derived Irregular Porous Graft Model

**Figure 3:**
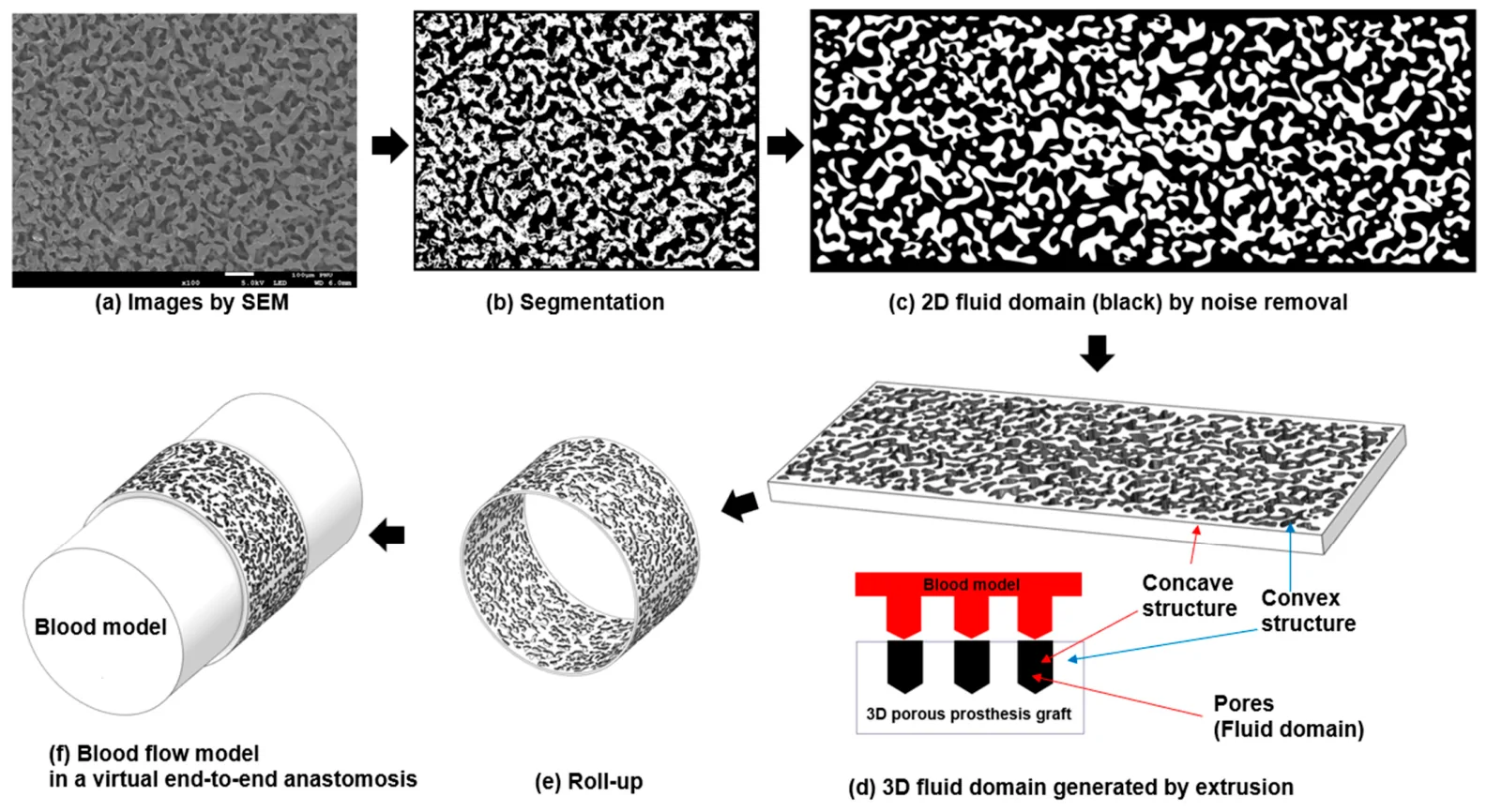
Workflow for reconstructing the irregular porous graft model (Model III) from a scanning electron microscopy (SEM) image. (**a**) Original SEM image. (**b**) Pore segmentation obtained by intensity thresholding at a value of 118. (**c**) Segmentation after removal of artifacts smaller than 200 pixels, representing the two-dimensional (2D) fluid domain within the pores. (**d**) Generation of a three-dimensional (3D) fluid domain by extruding the segmented 2D fluid domain to a thickness of 1.25 mm. (**e**) Wrapping of the extruded fluid domain into a cylindrical graft geometry while preserving the segmented pore morphology. (**f**) 3D blood flow model in a virtual end-to-end graft geometry (Model III) obtained by connecting the reconstructed porous graft to the mother vessel for CFD analysis.

The reconstructed irregular porous graft model (Model III) was generated from scanning electron microscopy (SEM) images reported in a previous study [17,26]. The SEM images had a spatial resolution of 1280 × 960 with an image scale of 1.07 pixels μm^−1^ (Figure 3a). Pores appear black, whereas the graft material appears gray in Figure 3a. Image segmentation was performed using an in-house image-processing code based on intensity thresholding, in which pixels satisfying I(x, y) < 118 were classified as pore regions (black) corresponding to the fluid domain, whereas the remaining pixels were classified as solid material (white) (Figure 3b). To improve the segmentation quality, artifacts smaller than 200 pixels were removed (Figure 3c). The segmented two-dimensional (2D) fluid domain within the pores was extruded to a thickness of 1.25 mm, generating a three-dimensional (3D) planar fluid domain within the porous layer (Figure 3d). The resulting 3D fluid domain was subsequently wrapped into a cylindrical graft geometry while preserving the segmented pore morphology using the commercial software Rhino 3D version 6 (Robert McNeel & Associates, USA) (Figure 3e). The volume porosity (void volume fraction) of the reconstructed porous graft was calculated as the ratio of the pore-fluid volume to the sum of the pore-fluid and solid volumes. The calculated volume porosity was 59%. The reconstructed fluid domain within the porous graft was then merged with the mother-vessel fluid domain to generate the three-dimensional (3D) blood flow model used for CFD simulations (Figure 3f).

### 2.3. Mesh Generation and Independence Analysis

**Figure 4:**
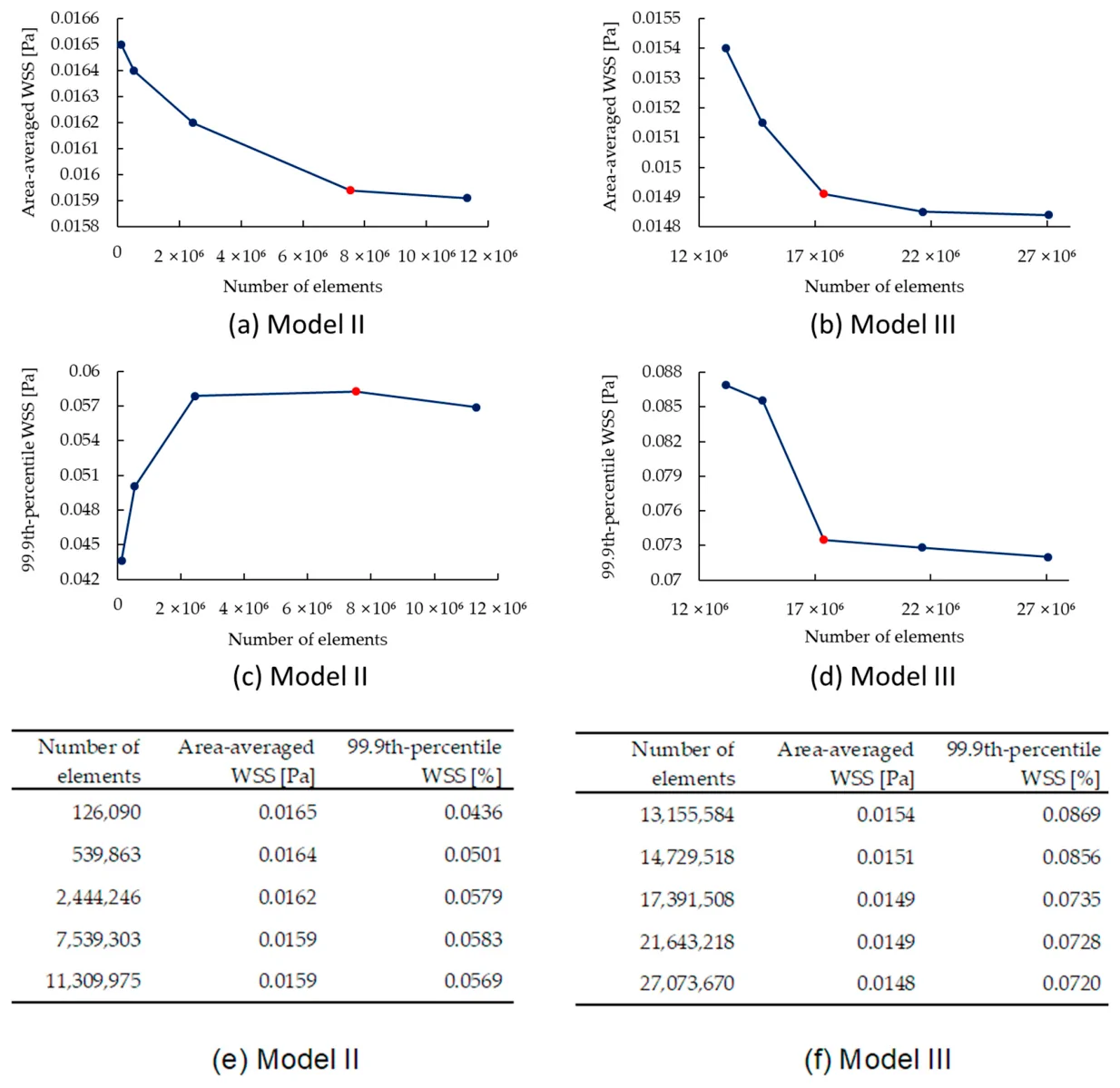
Mesh-independence tests based on wall shear stress. (**a**) Variation in area-averaged wall shear stress with the number of mesh elements for Model II. (**b**) Variation in area-averaged wall shear stress with the number of mesh elements for Model III. (**c**) Variation in 99.9th-percentile wall shear stress with the number of mesh elements for Model II. (**d**) Variation in 99.9th-percentile wall shear stress with the number of mesh elements for Model III. (**e**) Mesh-independence tests for Model II. (**f**) Mesh-independence tests for Model III. The selected meshes are indicated by red circles in (**a**–**d**).

For three blood models, computational domains were discretized using unstructured tetrahedral meshes generated with commercial software ANSYS Fluent Meshing v.2023 (ANSYS Inc., Canonsburg, PA, USA) (Figure 2c,d). Three prism inflation layers were generated adjacent to the vessel wall to improve the resolution of the near-wall boundary layer. To evaluate mesh independence, different mesh resolutions were examined at the pore base by gradually increasing the number of elements. The mesh-independent solution was considered to be achieved when the difference in the area-averaged wall shear stress (WSS) and the 99.9th-percentile wall shear stress between two successive mesh resolutions was less than 1% (Figure 4). Based on the mesh-independence tests, the selected meshes contained approximately 3.80 × 10^5^ elements for Model I, 7.54 × 10^6^ elements for Model II, and 1.74 × 10^7^ elements for Model III (Figure 4). The minimum orthogonal quality of the selected meshes was 0.20 for Model I, 0.13 for Model II, and 0.05 for Model III. And maximum skewness of the selected meshes was 0.363, 0.464 and 0.896 for Models I, II, and III, respectively. All meshes satisfied the recommended mesh quality criteria for CFD simulations.

### 2.4. Governing Equations and Numerical Methods

The CFD (computational fluid dynamics) simulations were conducted using a commercial solver (ANSYS Fluent software, v. 2023). This computational approach relied on the finite volume method, incorporating finite difference discretizations for the solution of the governing equations. For the numerical simulation, continuity equations (Equation (1)) and Navier–Stokes equations (Equation (2)) were solved.

∇ · ***u*** = 0 (1)

(2) $$(u\;\cdot \;\nabla )u=-\frac{1}{\rho }\nabla p+\frac{\mu }{\rho }\nabla^{2}u$$

Here, ***u***, *ρ*, and μ are the velocity vector, fluid density, and dynamic viscosity, respectively. A pressure-based solver was employed with the coupled pressure–velocity algorithm. Pressure was discretized using a second-order scheme, whereas the momentum equations were discretized using the second-order upwind scheme. The convergence criterion for all residuals was set to 1 × $10^{-6}$.

A fully developed flow with a Reynolds number (${Re}_{peak}$) of 500 was imposed as the inlet boundary condition. The flow was considered incompressible, Newtonian, laminar steady flow. Blood in the artery, e.g., aorta and coronary artery, is considered a Newtonian fluid. The wall shear stress is calculated as a result of the velocity gradient near the wall, i.e., no-slip condition.

### 2.5. Boundary Conditions and Fluid Properties

Blood was assumed to be an incompressible Newtonian fluid with a density of 1050 kg $m^{-3}$ and a dynamic viscosity of 0.00408 kg $m^{-1}s^{-1}$. The vessel wall was assumed to be rigid with a no-slip boundary condition. And the flow was considered laminar steady flow. A fully developed parabolic velocity profile corresponding to a Reynolds number of 500, defined using the peak inlet velocity, was prescribed at the inlet. The peak inlet velocity at the inlet centerline was 0.0531 ${ms}^{-1}$, and mean inlet velocity was 0.02655 ${ms}^{-1}$. The inlet velocity profile is described by Equation (3).

(3) $$u(r)=U_{peak}(1-({\frac{r}{R})}^{2})=2U_{mean}(1-({\frac{r}{R})}^{2})\;$$

where $U_{mean}$ and $U_{peak}$ are the mean inlet velocity and the peak inlet velocity, respectively. R is the inlet radius, and r is the radial distance from the centerline.

A zero-gauge-pressure boundary condition was imposed at the outlet. These boundary conditions were applied identically to Models I, II, and III to enable comparison of the effects of pore morphology on the flow characteristics.

## 3. Results

We consider the steady flow at ${Re}_{peak}\;$= 500 in virtual end-to-end anastomosis using three types of prosthetic graft models: (i) a non-porous prosthesis (Model I), (ii) a regular square-prism porous prosthesis (Model II) and (iii) an SEM-reconstructed irregular porous prosthesis (Model III). We simulated and compared hemodynamic characteristics of these three models, including velocity, pressure, and wall shear stress (WSS), and the flow streamline.

**Figure 5:**
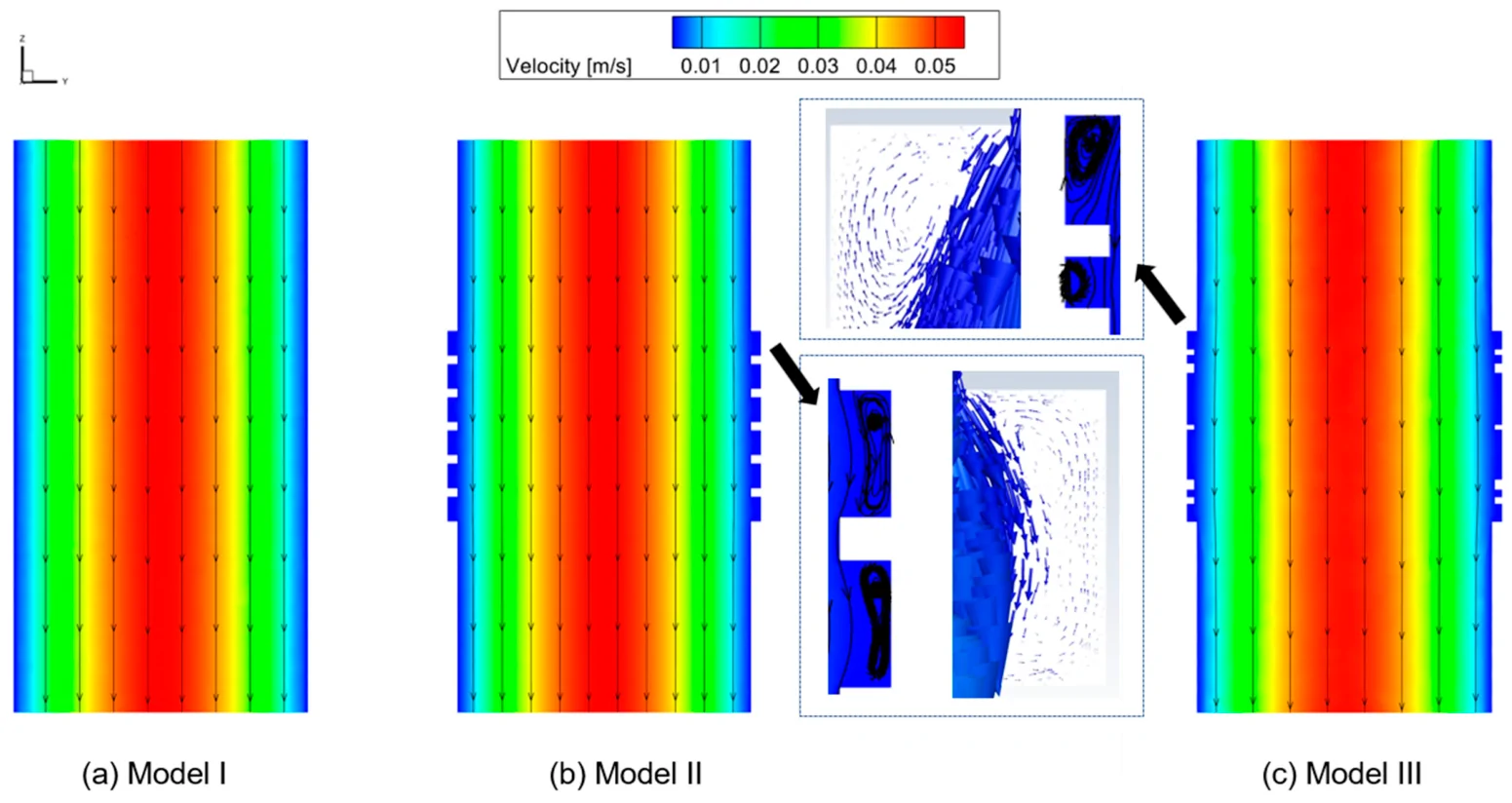
Velocity distributions with streamlines in three models. (**a**) Model I. (**b**) Model II: recirculations in pores and velocity vectors. (**c**) Model III: recirculations in pores and velocity vectors.

**Figure 6:**
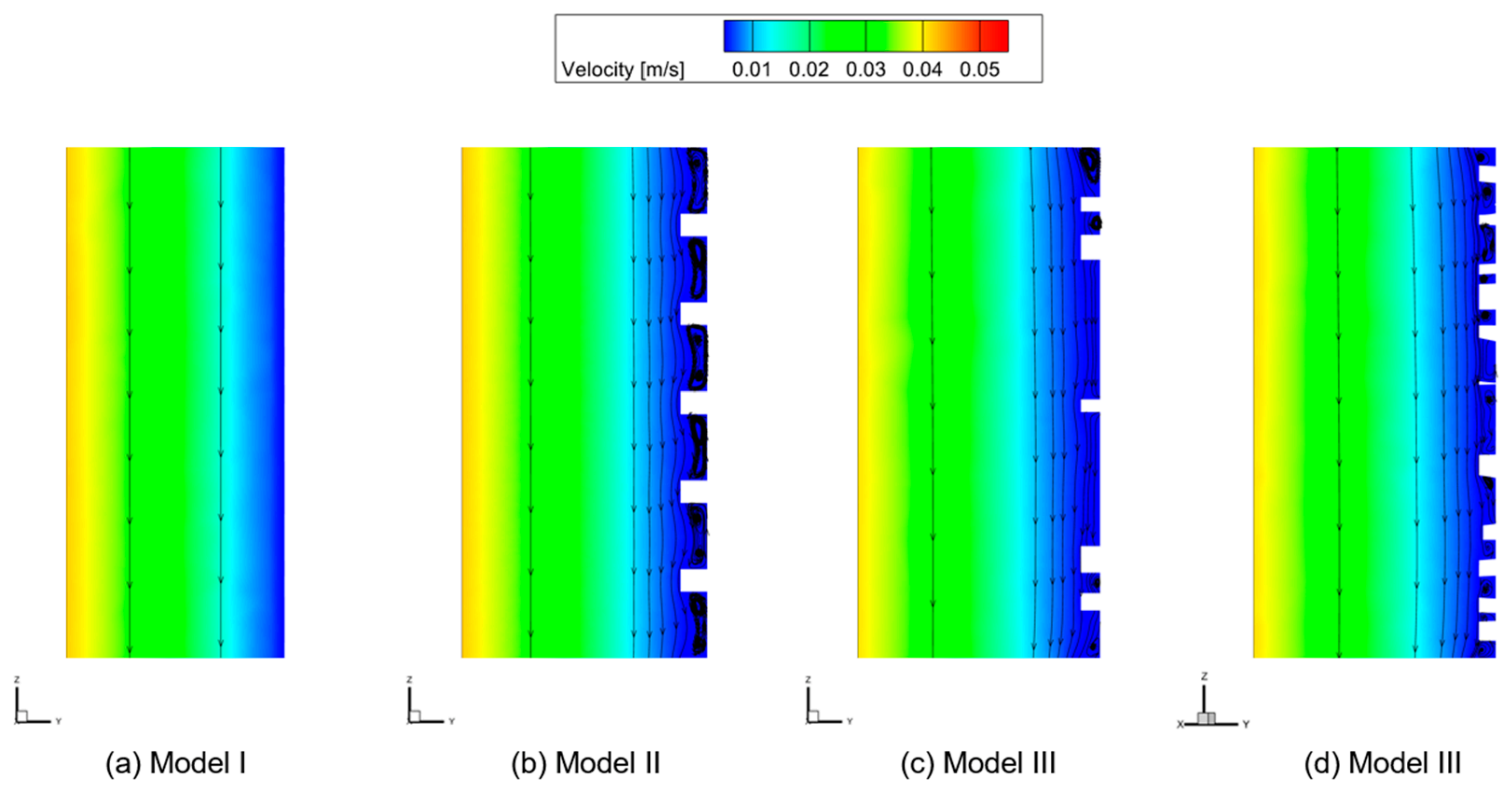
Velocity distributions with streamlines near pores in three models. (**a**) Model I without pores without recirculations. (**b**) Model II: regular recirculation patterns in pores. (**c**) Model III: irregular recirculations within the irregular pores. (**d**) Model III from a different perspective: irregular recirculations within the irregular pores viewed from a different perspective.

**Figure 7:**
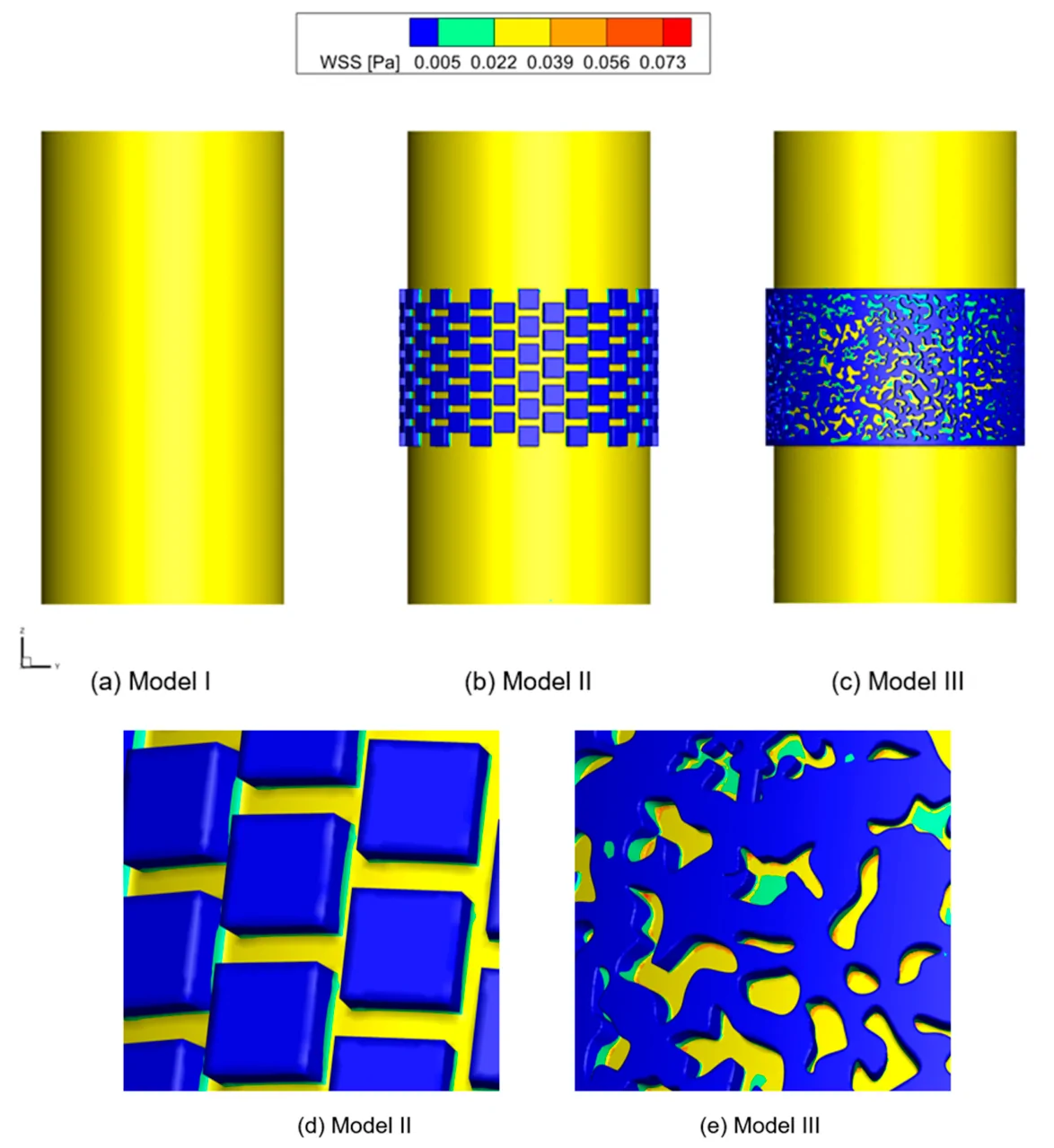
Wall shear stress distributions. (**a**) Model I, (**b**) Model II, and (**c**) Model III. Enlarged views of WSS distributions in the pore regions of (**d**) Model II and (**e**) Model III.

**Figure 8:**
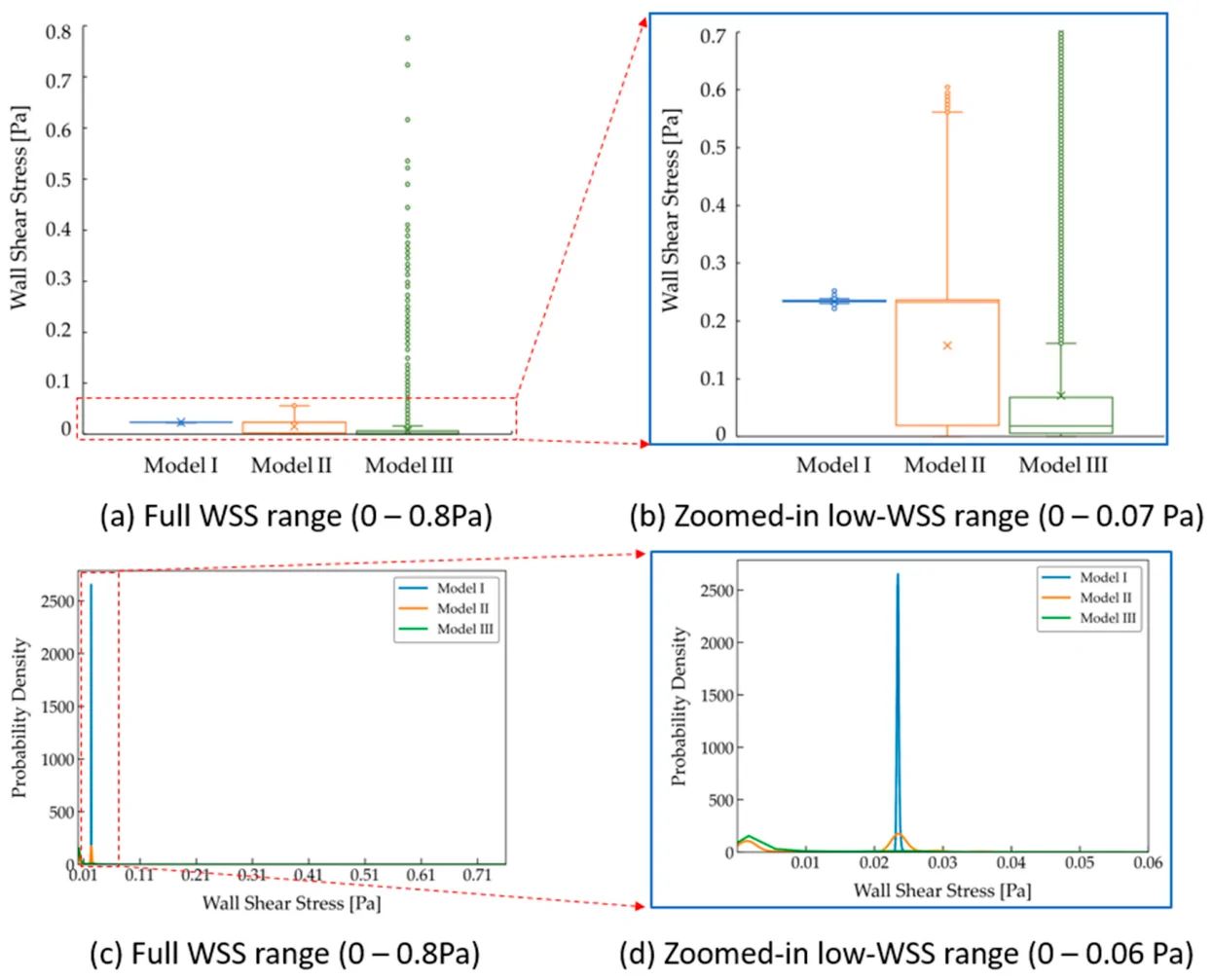
Comparison of wall shear stress (WSS) distributions among the three graft models. Box plots: The box denotes the interquartile range (IQR), the horizontal line the median, and “×” the mean; whiskers extend to 1.5 × IQR, and circles denote outliers. (**a**) Box plots of the full WSS range (0–0.8 Pa) for Models I–III, showing the overall WSS distribution and localized high-WSS outliers. (**b**) Zoomed-in box plots of the low-WSS range (0–0.07 Pa), illustrating the median, interquartile range (IQR), whiskers, and outliers; the × symbol denotes the mean WSS. (**c**) Kernel density estimation (KDE) curves of the full WSS range (0–0.8 Pa) for Models I–III. (**d**) Zoomed-in KDE curves of the low-WSS range (0–0.06 Pa), highlighting differences in the probability-density distributions among the three models.

**Figure 9:**
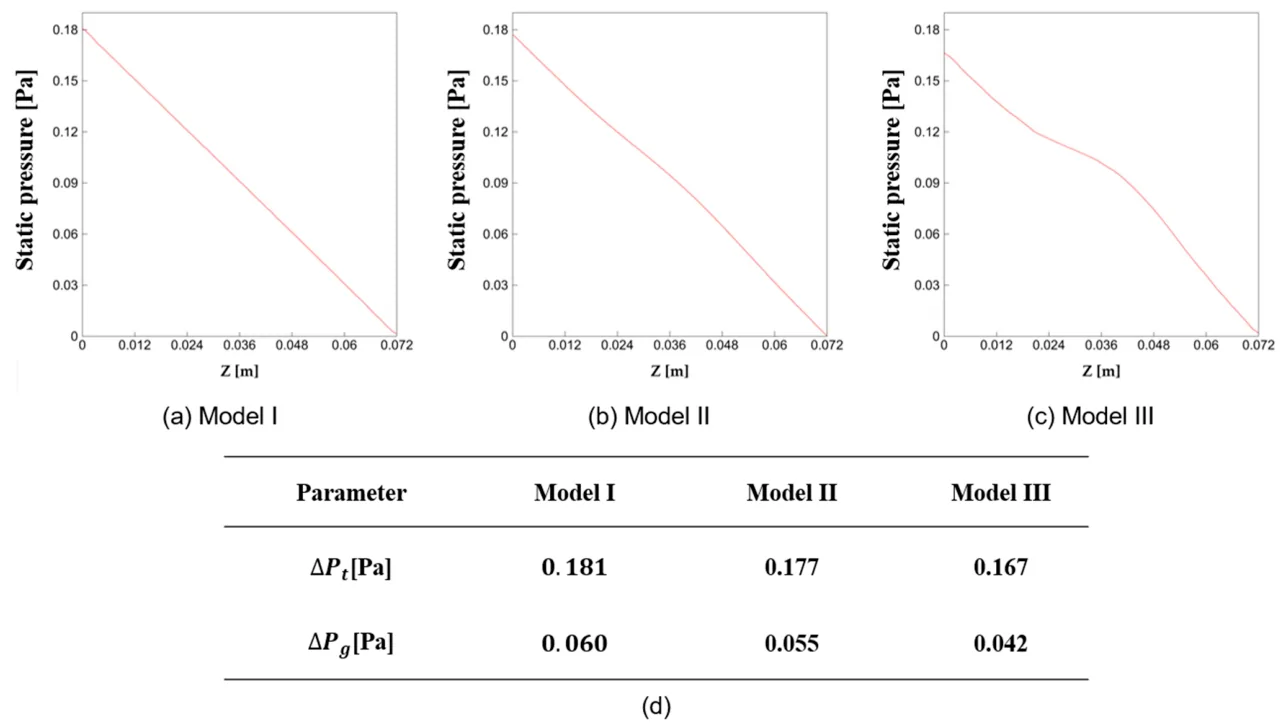
Centerline static-pressure distributions along the axial direction from the inlet to the outlet, indicated by red solid lines, for (**a**) Model I, (**b**) Model II, and (**c**) Model III. (**d**) Table comparing the centerline static-pressure difference across the entire computation domain (ΔPt) (z = 0~0.072 m) and the centerline static-pressure difference across the porous graft section (ΔPg) (z = 0.024~0.048 m) for Models I–III.

Model I, characterized by a non-porous rigid wall, exhibited fully developed laminar flow characteristics in the straight arterial model. The blood flow in Model I exhibited velocity distributions typical of fully developed pipe flow, with the highest velocity at the centerline at 0.0531 ${ms}^{-1}\;$ and progressively lower velocities toward the vessel wall (Figure 5a). There were no recirculation zones on the non-porous wall, in contrast to Models II and III (Figure 6a). Accordingly, the wall shear stress (WSS) distribution was circumferentially uniform along the graft wall, with a value of approximately 0.0235 Pa (Figure 7a and Figure 8 and Table 1 and Table 2). The box plots and kernel density estimation (KDE) curves in Figure 8 further confirm the highly uniform WSS distribution in Model I. The full-range plots demonstrate the absence of high-WSS outliers, whereas the zoomed-in views clearly show that both the mean and median WSS values are concentrated around 0.023 Pa with minimal spatial variation throughout the graft. The centerline static pressure decreased almost linearly from the inlet to the outlet of the entire computational domain, resulting in a centerline static-pressure difference across the entire computational domain (ΔPt) of 0.181 Pa and a centerline static-pressure difference across the prosthetic graft section (ΔPg) of 0.060 Pa (Figure 8a,d and Figure 9 and Table 1). These results are in excellent agreement with the analytical Hagen–Poiseuille solution, with relative errors of 0.5% for the centerline static-pressure difference, 0.4% for wall shear stress, and 0.7% for flow rate (Table 1), confirming the accuracy of the numerical solver.

In Model II, where a regular square-prism porous wall was employed, the blood flow simulations demonstrated that the presence of the regular porous structure significantly modified the local blood flow dynamics. The porous structure caused the formation of recirculating flow near the porous wall, particularly within and around the square-prism pores (Figure 5b and Figure 6b). The recirculating flows generated localized low-velocity regions in the porous area (Figure 5b). Although the porous structure altered the local flow field, the overall velocity distribution remained similar to that of Model I. The WSS distribution exhibited a regular pattern reflecting the periodic arrangement of the square-prism pores (Figure 7b,d and Figure 8). The mean and median WSS values were 0.0159 Pa and 0.0232 Pa, respectively, whereas the maximum WSS reached 0.0606 Pa (Table 2). The minimum WSS value of Model I (0.022 Pa) was adopted as a reference threshold for defining relatively low WSS. Based on this criterion, 34.58% of the graft surface area in Model II exhibited WSS values below 0.022 Pa (Table 2). As shown in Figure 7 and Figure 8, these low-WSS regions (green and blue) were predominantly distributed within and around the porous structures, indicating that the porous morphology promoted the formation of localized low-WSS regions. The full-range box plot (Figure 8a) shows that only a limited number of localized high-WSS outliers were present in Model II. In contrast, the zoomed-in box plot (Figure 8b) demonstrates that the median WSS remained comparable to that of Model I, whereas the interquartile range and whiskers are noticeably broader, indicating greater spatial variability in WSS. Similarly, the full-range KDE curve (Figure 8c) confirms that high-WSS values were relatively uncommon, while the zoomed-in KDE curve (Figure 8d) reveals a broader probability-density distribution with an increased proportion of low-WSS values. Similarly to Model I, the centerline static pressure decreased almost linearly in Model II, resulting in a centerline static-pressure difference across the entire computational domain (ΔPt) of 0.177 Pa and a centerline static-pressure difference across the prosthetic graft section (ΔPg) of 0.055 Pa (Figure 9b,d).

In Model III, which was reconstructed from segmented SEM images to preserve the realistic irregular pore morphology, the reconstructed fluid domain within the porous graft produced marked spatial variations in the local flow field. Disturbed flow and irregular recirculation patterns were observed within and around some of the pores, whereas recirculation was not evident in all pores (Figure 5c and Figure 6c,d). Similarly, the WSS distribution exhibited pronounced spatial heterogeneity over the irregular porous surface (Figure 7c,e). The mean and median WSS values were 0.0149 Pa and 0.00182 Pa, respectively, whereas the maximum WSS reached 0.7759 Pa (Table 2). Model III exhibited the lowest mean and median WSS among the three models (Figure 8a and Table 2). Nevertheless, localized high-WSS regions were observed at the sharp edges of the irregular pores, where the flow accelerated locally. In addition, 44.37% of the graft surface area exhibited WSS values below 0.022 Pa (Table 2 and Figure 7). These low-WSS regions were broadly distributed within and around the irregular porous structures. The full-range box plot (Figure 8a) demonstrated that Model III exhibited the broadest WSS distribution among the three models, including numerous localized high-WSS outliers extending to the maximum WSS value of 0.7759 Pa. In contrast, the zoomed-in box plot (Figure 8b) showed that the majority of WSS values were concentrated within the low-WSS range despite these localized extreme values. Similarly, the full-range KDE curve (Figure 8c) illustrated the complete WSS distribution, whereas the zoomed-in KDE curve (Figure 8d) highlighted a pronounced shift in the probability density toward the low-WSS region near zero. These results demonstrate that the WSS distribution in Model III was characterized by predominantly low WSS over most of the graft surface together with localized regions of markedly elevated WSS at the sharp pore edges. As demonstrated by the full-range and zoomed-in box plots and KDE curves (Figure 8), these localized high-WSS regions occupied only a small fraction of the graft surface, whereas the majority of the surface was exposed to low WSS.

The pressure distribution also differed from those of Models I and II. The centerline static pressure decreased continuously along the axial direction, yielding a centerline static-pressure difference across the entire computation domain (from the inlet of the proximal mother vessel to the outlet of the distal mother vessel) (∆Pt) of 0.167 Pa and a centerline static-pressure difference across the prosthetic graft (∆Pg) of 0.042 Pa (Figure 9c,d). The centerline static-pressure differences across both the prosthetic graft and the entire computational domain were lower in Model III than in Models I and II. Unlike the nearly linear pressure distributions observed in Models I and II, Model III exhibited a non-uniform axial pressure gradient along the graft, reflecting spatial variations in pressure associated with the irregular pore morphology.

## 4. Discussion

The results of our study highlight the intricate influence of prosthetic graft pore morphology on blood flow dynamics in a virtual end-to-end anastomosis scenario. The present study compared the hemodynamic characteristics of non-porous, regular porous, and SEM-reconstructed irregular porous prosthetic graft models under steady laminar flow at ${Re}_{peak}$ = 500. The results demonstrate that pore morphology substantially influenced local flow recirculation, pressure distribution, and wall shear stress patterns, even though the overall axial flow remained largely preserved. In particular, the regular porous model produced periodic recirculation and WSS patterns corresponding to the repeated square-prism pore arrangement, whereas the SEM-reconstructed irregular porous model generated markedly heterogeneous local flow and WSS distributions. These findings indicate that not only the presence of pores but also their morphology, spatial arrangement, and edge geometry are important determinants of the local hemodynamic environment.

Model I served as the reference model and was used to verify the numerical approach. This result shows good agreement with previous studies [26]. And the computational centerline static-pressure difference, WSS, and flow rate also agree closely with the analytical Hagen–Poiseuille solution, with relative errors below 1%. This agreement confirms that the numerical model accurately reproduced fully developed laminar pipe flow under the present conditions. The absence of recirculation and the nearly uniform circumferential WSS distribution in Model I are also consistent with the expected behavior of fully developed flow in a straight circular conduit. Therefore, the differences observed in Models II and III can primarily be attributed to the presence and morphology of the porous surface structures rather than to numerical error.

In Model II, the periodically arranged square-prism pores altered the local flow field by producing recirculation zones. These recirculation zones resulted in slower-moving flows within the porous area. However, the pores did not significantly alter the overall velocity distribution outside the pores, and the bulk flow pattern remained similar to that of Model I. Because the pore geometry and spacing were regular, these flow disturbances were also spatially repetitive. This behavior was reflected in the WSS distribution, which exhibited a periodic pattern corresponding to the arrangement of the square-prism pores. Although the median WSS of Model II remained close to that of Model I, the mean WSS was lower, and 34.58% of the graft surface area exhibited WSS values below 0.022 Pa. This difference between the mean and median indicates that localized low-WSS regions occupied a substantial portion of the porous surface, while comparatively higher WSS values remained near selected pore edges or exposed wall regions. Thus, even a geometrically regular porous structure introduced a considerably more heterogeneous near-wall hemodynamic environment than the non-porous graft.

The most pronounced hemodynamic differences were observed in Model III, which was reconstructed from segmented SEM images to preserve the realistic irregular pore morphology. Compared with the regular porous geometry of Model II, the irregular porous structure produced greater spatial variations in the local flow field. Recirculation was observed within and around some pores but was not present in all pores, indicating that local flow behavior depended on variations in pore geometry. Consequently, low-velocity and recirculating regions were distributed irregularly over the porous surface rather than repeating periodically as in Model II. These findings demonstrate that idealized periodic pore models cannot fully reproduce the local hemodynamic characteristics generated by realistic porous microstructures. Although regular porous models are valuable for investigating the influence of individual geometric parameters, SEM-based reconstruction provides a more realistic representation of the spatial variability in local flow patterns.

The irregular porous morphology adopted in Model III was reconstructed from a previously developed three-dimensional (3D) templated vascular graft that had been mechanically characterized and evaluated in vivo [26]. The graft exhibited mechanical properties comparable to or better than those of commercially available vascular grafts and demonstrated favorable performance in a rat implantation model [26]. Although the previous study did not include quantitative hemodynamic measurements for direct CFD validation, the present CFD analysis complements the experimental study by providing detailed local flow characteristics associated with the irregular porous structure that cannot be readily obtained through experiments alone.

The pressure results further distinguished Model III from Models I and II. The centerline static-pressure differences across both the entire computational domain and the prosthetic graft section were lowest in Model III. In addition, the pressure distribution in Model III was less uniform than those in Models I and II. The non-uniform axial pressure gradient suggests that local pressure variations along the centerline were influenced by the irregular pore morphology. Accordingly, the lower centerline static-pressure differences should be interpreted as comparative indicators of pressure variation along the vessel axis rather than as comprehensive measures of global hydraulic or mechanical-energy loss. Rather, Model III exhibited lower centerline static-pressure differences while simultaneously showing stronger local variations in velocity, pressure gradient, and near-wall flow.

The WSS distribution in Model III was the most heterogeneous among the three models. It exhibited the lowest mean and median WSS, while simultaneously producing the highest maximum WSS and numerous high-WSS outliers. The very low median value indicates that a large proportion of the graft surface was exposed to low WSS. This interpretation is consistent with the finding that 44.37% of the surface area had WSS values below 0.022 Pa, which was greater than the corresponding area in Model II. In contrast, the highest WSS values were confined to localized regions, primarily near sharp pore edges where the flow accelerated. These localized peaks occurred at sharp edges introduced by the idealized representation of the reconstructed pore geometry and may therefore be influenced by localized numerical stress concentrations in addition to the underlying flow physics. Accordingly, the maximum WSS should be interpreted together with the overall WSS distribution and complementary statistical descriptors, rather than as an independent indicator of the local hemodynamic environment. Consequently, the mean WSS remained substantially higher than the median despite the predominance of low WSS values over much of the graft surface. These results show that a single representative WSS value, such as the mean alone, is insufficient to characterize the hemodynamic environment of an irregular porous graft. The median, maximum, standard deviation, low-WSS surface area, and spatial WSS distribution should be considered together.

From a graft-design perspective, the comparison between Models II and III suggests that porosity alone does not fully determine the local hemodynamic response. Although both porous models had porous wall structures, their flow and WSS characteristics differed considerably because of differences in pore regularity and local geometry. The regular porous model generated predictable and repeated recirculation patterns, whereas the irregular model created a broader range of local hemodynamic conditions. In particular, the coexistence of extensive low-WSS regions and highly localized high-WSS regions in Model III may be important when evaluating surface designs. Reducing sharp pore edges and excessively irregular geometrical transitions may help limit extreme WSS concentrations, whereas controlling pore size, spacing, and orientation may reduce the extent of low-velocity and low-WSS regions. However, these interpretations should be regarded as design-related hemodynamic considerations rather than direct evidence of biological performance.

The present study has several limitations. First, steady laminar flow was assumed to isolate the effects of pore geometry while minimizing additional variables introduced by pulsatile flow, whereas physiological arterial flow is pulsatile and may produce time-dependent recirculation, flow separation, and oscillatory WSS. Accordingly, future work should incorporate physiologically realistic pulsatile flow conditions to evaluate time-dependent hemodynamic parameters, including time-averaged wall shear stress (TAWSS), oscillatory shear index (OSI), and relative residence time (RRT). Recent computational studies have demonstrated the importance of these transient WSS-based metrics for evaluating morphology-dependent cardiovascular hemodynamics. Liu et al. reported that TAWSS, OSI, and RRT were effective indicators for identifying regions susceptible to in-stent thrombosis following branched endovascular repair [10], whereas Sarkhosh et al. showed that arterial morphology substantially influenced TAWSS distributions across a large population of idealized geometries, demonstrating that even subtle geometric variations can significantly alter local wall shear stress patterns [12]. Although the present study was limited to steady-state simulations and therefore did not evaluate TAWSS, OSI, or RRT, the observed pore-scale WSS heterogeneity complements rather than replaces transient hemodynamic assessment. The present results provide detailed insight into the local flow disturbances generated by realistic porous microstructures and establish a foundation for future investigations under physiologically realistic pulsatile flow conditions. Second, blood was modeled as a Newtonian fluid with a constant viscosity. Although this assumption is generally acceptable for large-vessel flow under relatively high-shear conditions, it does not account for the shear-thinning behavior arising from red blood cell deformation and aggregation, particularly in low-shear regions within the porous structures. Consequently, local hemodynamics in regions of flow stagnation and recirculation may differ from those predicted by the present model. Third, the graft and mother-vessel walls were modeled as rigid, and therefore, wall deformation, compliance mismatch, and solid–fluid interaction were not considered. Fourth, the present study employed an idealized vascular geometry rather than reproducing a specific anatomical artery. The vessel diameter was selected to enable controlled comparisons among the three graft configurations under dynamically similar flow conditions. In addition, only one flow condition and one reconstructed irregular porous morphology were analyzed. Consequently, the results may depend on the Reynolds number, vessel size, pore size, porosity, surface thickness, pore connectivity, and reconstruction accuracy. Furthermore, although Models II and III were designed to have the same volumetric porosity (59%), they simultaneously differed in pore-size distribution, connectivity, spacing, orientation, edge geometry, and local surface characteristics. Therefore, the present comparison should be interpreted as an evaluation of the combined effects of pore architecture under matched porosity conditions rather than as an isolated assessment of pore shape alone. Accordingly, the present findings should be interpreted as a comparative evaluation of pore-scale hemodynamics rather than as vessel-specific predictions. Fifth, the low-WSS threshold of 0.022 Pa was defined using the minimum WSS of Model I as a relative reference for comparison among the three models and should not be interpreted as a universal physiological threshold. Sixth, although the computational model was validated against the analytical Hagen–Poiseuille solution and the irregular porous morphology was reconstructed from a previously developed graft that had been mechanically characterized and evaluated in vivo, direct experimental validation of the predicted flow fields using techniques such as particle image velocimetry (PIV) or benchtop flow-loop experiments was not performed. Therefore, the pore-scale hemodynamic predictions presented in this study should be interpreted within the limitations of the computational framework. Seventh, the porous regions were treated as impermeable surface geometries, and fluid transport through the graft material was not modeled. Finally, the present study focused exclusively on the hemodynamic effects of pore morphology and did not consider the biological responses and physicochemical interactions associated with graft implantation. Therefore, the present findings should be interpreted as results of a hemodynamic evaluation rather than a comprehensive assessment of graft biocompatibility. Future studies will incorporate non-Newtonian blood models, direct experimental validation using PIV measurements and benchtop flow-loop experiments, pulsatile flow, fluid–structure interaction analyses, and integrated biological evaluations to improve both the physiological realism of the computational model and the assessment of the biocompatibility of porous prosthetic grafts.

Despite these limitations, the present results demonstrate that SEM-based reconstruction provides additional hemodynamic information that cannot be fully captured by an idealized regular porous model. The irregular porous graft exhibited lower centerline static-pressure differences but substantially greater spatial heterogeneity in recirculation and WSS, including extensive low-WSS regions and localized high-WSS concentrations at sharp pore edges. These findings emphasize the need to evaluate both centerline pressure variations and local near-wall hemodynamics when designing and assessing porous prosthetic graft surfaces. Future studies should extend the present framework to pulsatile flow, compliant walls, multiple SEM-derived geometries, and experimental validation to determine how the combined effects of pore architecture under matched porosity conditions influence hemodynamics within prosthetic grafts under more physiologically realistic conditions.

## 5. Conclusions

This study investigated the effects of pore morphology on blood flow characteristics in prosthetic vascular grafts by comparing three models: a non-porous graft, a regular square-prism porous graft, and an SEM-reconstructed irregular porous graft. The numerical model was first validated against the analytical Hagen–Poiseuille solution, demonstrating excellent agreement in centerline static-pressure difference, wall shear stress (WSS), and flow rate.

Compared with the non-porous graft, both porous grafts generated local recirculation zones and low-velocity regions around the pores, resulting in more heterogeneous WSS distributions. The regular porous graft produced periodic flow and WSS patterns corresponding to the regular pore arrangement while largely preserving the overall velocity distribution. In contrast, the SEM-reconstructed irregular porous graft generated irregular recirculation patterns, a non-uniform pressure distribution, and the greatest spatial variability in WSS. Although Model III exhibited the lowest mean and median WSS, it also produced the highest localized WSS at sharp pore edges and the largest surface area exposed to low WSS, demonstrating the coexistence of extensive low-WSS regions and localized high-WSS concentrations.

These findings demonstrate that pore morphology, rather than porosity alone, plays an important role in determining local hemodynamic characteristics. Furthermore, the SEM-based reconstruction approach captured realistic spatial variations in flow and WSS that could not be fully reproduced by an idealized regular porous model. The proposed computational framework provides a useful tool for evaluating porous graft surface designs and may contribute to the future optimization of prosthetic vascular grafts by incorporating both hemodynamic performance and biological considerations.

## Figures and Tables

**Table 1:** Comparison of the analytical Hagen–Poiseuille solution and computational results for fully developed laminar flow in Model I, including centerline pressure difference, wall shear stress, flow rate, and relative errors.

Parameter	Equation	Analytical Solutions	CFD	Error [%]
Pressure difference [Pa]	$∆P=\frac{32\mu UL}{D^{2}}$	0.182	0.181	0.5
Wall shear stress [Pa]	$\tau_{w=}\frac{8\mu U}{D}$	0.0234	0.0235	0.4
Flow rate [$m^{3}s^{-1}]$	$Q=\frac{\pi \mu }{4}U$	$2.85\times 10^{-5}$	$2.83\times 10^{-5}$	0.7

**Table 2:** Statistical comparison of wall shear stress (WSS) distributions for Models I–III, including the mean, median, minimum, maximum, and standard deviation (SD), and the surface area with WSS below 0.022 Pa.

Model	Mean WSS [Pa]	Median WSS [Pa]	Min WSS [Pa]	Max WSS [Pa]	WSS SD [Pa]	Surface Area with WSS < 0.022 Pa [%]
Model I	0.0235	0.0234	0.0222	0.0254	0.0001820	0.00
Model II	0.0159	0.0232	$2.0\times 10^{-6}$	0.0606	0.0120093	34.58
Model III	0.0149	0.00182	$1.1\times 10^{-9}$	0.7759	0.0121743	44.37

## Data Availability

The data supporting the findings of this study are available from the corresponding author upon reasonable request.
